# Determinants of caesarean section in Bangladesh: Cross-sectional analysis of Bangladesh Demographic and Health Survey 2014 Data

**DOI:** 10.1371/journal.pone.0202879

**Published:** 2018-09-12

**Authors:** Mohammad Masudur Rahman, Mohammad Rifat Haider, Md. Moinuddin, Ahmed Ehsanur Rahman, Shakil Ahmed, M. Mahmud Khan

**Affiliations:** 1 Maternal and Child Health Division (MCHD), International Centre for Diarrhoeal Disease Research, Bangladesh (icddr,b), Dhaka, Bangladesh; 2 Department of Health Promotion, Education and Behavior, Arnold School of Public Health, University of South Carolina, Columbia, South Carolina, United States of America; 3 Department of Health Services Policy and Management, Arnold School of Public Health, University of South Carolina, Columbia, South Carolina, United States of America; 4 Department of Public Health and Informatics, Jahangirnagar University, Savar, Dhaka, Bangladesh; 5 Department of Statistical Science, University of Padova, Italy; 6 The World Bank, Dhaka, Bangladesh; BRAC, BANGLADESH

## Abstract

**Background:**

Caesarean section (CS) has been on the rise worldwide and Bangladesh is no exception. In Bangladesh, the CS rate, which includes both institutional and community-based deliveries, has increased from about 3% in 2000 to about 24% in 2014. This study examines the association of reported complications around delivery and socio-demographic, healthcare and spatial characteristics of mothers with CS, using data from the latest Bangladesh Demographic and Health Survey (BDHS).

**Methods:**

The study is based on data from the 2014 BDHS. BDHS is a nationally representative survey which is conducted periodically and 2014 is the latest of the BDHS conducted. Data collected from 4,627 mothers who gave birth in health care institutions in three years preceding the survey were used in this study.

**Results:**

Average age of the mothers was 24.6 years, while their average years of schooling were 3.2. Factors like mother being older, obese, residing in urban areas, first birth, maternal perception of large newborn size, husband being a professional, had higher number of antenatal care (ANC) visits, seeking ANC from private providers, and delivering in a private facility were statistically associated with higher rates of CS.

**Conclusions:**

Bangladesh health system urgently needs policy guideline with monitoring of clinical indications of CS deliveries to avoid unnecessary CS. Strict adherence to this guideline, along with enhance knowledge on the unsafe nature of the unnecessary CS can achieve increased institutional normal delivery in future; otherwise, an emergency procedure may end up being a lucrative practice.

## Background

Caesarean section (CS) is a surgical procedure to reduce complications associated with childbirth. CS has witnessed a rising trend during the last three decades globally and the current rates greatly vary across different countries in the world. In high-income countries CS rates seem to have risen from around 4% to 5% in 1970 to 20% to 22% in 2001 [1]. According to World Health Organization (WHO) 2010 report, the CS rate was the highest in Brazil (45.9%) and was probably the lowest in countries like India and Pakistan, about 7 to 8% [2]. During the last three decades the CS rates were lower than 10% in 52 countries, from 10% to 15% in 14 countries and above 15% in 69 countries of the world, though WHO in 1985 stated that the CS rates for any region should not be any higher than 10–15% [2, 3].

CS is a life-saving intervention for both the mother and unborn child. However, unnecessary CS deliveries are harmful for health and wellbeing of both mothers and the children [4, 5]. CS also increases the chance of having preterm or early term babies [6] and even newborn deaths [7]. In some settings it was found that maternal mortality can be as high as 2–4 times greater for CS than normal vaginal deliveries [8]. Comparison of health status of mothers using EuroQoL-5D indicates that health condition of mothers who had undergone CS deliveries was poorer than the health status of the mothers who had normal vaginal delivery [9]. Adoption of CS delivery is justifiable for a number of well-defined maternal indications including severe antepartum haemorrhage, major cephalo-pelvic disproportion, abnormal fetal presentation etc. [10]. Another approach to identify potential CS cases is to use Robson classification system. Robson classification proposed a system that categorized women into 10 groups based on their obstetric characteristics (parity, previous CS, gestational age, onset of labour, fetal presentation and number of fetuses). This classification, if successfully incorporated in the routine clinical practice of any country can help to study rates in homogeneous groups of population and act as a tool to monitor CS rates. Hence, the Robson criteria can be used as a tool to help reduce CS rates. [11–13].

In Bangladesh, the CS rate has increased more than eight-fold, from 2.7% in 2000 to 24% in 2014 [14]. In the past few decades, a number of maternal health related strategies were adopted and implemented by the government of Bangladesh. In 1994, basic and comprehensive emergency obstetric care (EmOC) services were introduced in peripheral public sector health care facilities by the Ministry of Health and Family Welfare (MOH&FW) [15]. A demand side financing maternal health voucher scheme was introduced in 2007 to increase the utilization of maternal health care services by the poor [16]. These services and schemes were implemented with the objective of increasing institutional deliveries. These policies have improved maternal health by lowering maternal and infant deaths as well as morbidities [16]. At the same time, however, improved access to comprehensive EmOC may have led to rapid increase in CS deliveries as an unintended consequence. Considering the potential negative consequences of CS deliveries when conducted without any clinical indications, this paper aims to find out the determinants of CS in Bangladesh, which will help us in understanding the existing patterns and take course corrective measures by prioritizing determining factors for reducing unnecessary CS in Bangladesh.

## Methods

### Data source

We conducted secondary analysis of birth data from the 2014 Bangladesh Demographic and Health Survey (BDHS). BDHS is a nationally representative survey which is conducted periodically and 2014 is the latest of the BDHS conducted so far. The 2014 Bangladesh Demographic and Health Survey (2014 BDHS) was implemented under the authority of the National Institute of Population Research and Training (NIPORT), Ministry of Health and Family Welfare. The survey was conducted by Mitra and Associates from June to November 2014. Funding was provided by the United States Agency for International Development (USAID)/Bangladesh. ICF International provided technical assistance through The DHS Program, a USAID-funded project. The data set can be obtained from DHS program website upon authorization (https://dhsprogram.com/data/available-datasets.cfm). BDHS follows a multi-stage cluster sampling technique. In the first stage, 600 clusters (393 from rural and 207 from urban strata) were randomly selected. In the second stage, 30 households were randomly selected from each of the clusters and all ever-married 15–49 years old mothers in the selected households were interviewed. The survey in 2014 resulted in 17,866 completed interviews of ever-married mothers of age 15–49 years. Information on mothers who gave birth in health care institutions in three years preceding the survey has been used in this study (N = 4,627) [17]. This study was exempt from the ethical review approval because it uses publicly available de-identified data.

### Determinants

Following the framework proposed by Leone et. al. [18], we have grouped the factors associated with CS delivery into four broad categories, e.g., social network related factors, health care institution related factors, demographic and pregnancy related risk factors, and socio-economic factors (Table 1). Social network factors include the media exposure, i.e., whether the woman watched TV at least once a week [18]. Institutional factors include ownership of the facility where the delivery was conducted (public vs. private), number of antenatal care visits (none, 1–2, 3 or more than 3) at the institutions, and antenatal care received in private facilities (yes or no) [19]. Demographic and pregnancy risk factors include maternal age (15–19, 20–24, 25–29, 30–49 years), maternal height (less than 60 inches, and 60 inches or more), birth order (first, second, third or higher), maternal nutritional status (undernourished, normal, and obese), size of the baby (larger than average, average, and smaller than average). Among these variables maternal nutritional status is measured by body-mass index (BMI) where mothers with BMI less than 18.5 were identified as undernourished, 18.5 to less than 25.0 as normal, and 25.0 or higher as overweight. Size of the baby was determined from the maternal recall of baby’s weight at birth. Mothers were asked the question about their baby’s weight using a five point likert scale and the responses were later recoded into larger than average, average, and smaller than average categories [20].

**Table 1:** Classification of potential factors affecting caesarean section in Bangladesh.

Social network Factors	Institutional factors	Demographic risk factors/complexity of the birth	Socio-economic factors
Regularly watches TV at least once a week	Public vs. private facility	Multiple births	Household wealth
	Has had one or more antenatal care contacts	Size of baby	Maternal Education
	ANC received from private facilities	History of previous caesareans	Husband’s Education
		Mother’s height	Maternal employment status
		Age of mother	Husband’s Occupation
		Parity	Residence
		Age at first birth	

Socio-economic factors consist of maternal education, husband’s education (no education, primary, secondary, and bachelor and higher), mothers’ employment status (yes/no), husband’s occupation (farmer/worker, carpenter/mason/driver, professional, business, and none/others), household socioeconomic status based on wealth scores (poorest, poorer, middle income, richer, and richest), and residence (urban and rural). An interaction term between two categorical variables residence and delivery in private facilities has also been included in the analysis to test differential effect of deliveries conducted in private facilities on CS after controlling for location of household.

### Data analysis

Descriptive analysis was performed to have a view of the characteristics of the study participants. Bivariate analysis was also done among the institutional deliveries (caesarean section or normal delivery) under the purview of the study. Chi-square test was performed to see any significant difference among the categories of each variable with the dependent variable of nature of delivery.

We have also looked at the reasons of choosing CS cited by the respondents based on the person who took the final decision of CS, either doctor or patient. Timing of the decision taking on performing CS was also analyzed. Timing was categorized into on the day of delivery, day before delivery, 2–7 days before the delivery, 8–30 days before the delivery, and more than 30 days before the delivery.

Multivariate analysis was done using a mixed effect multilevel model with the outcome variable being the type of delivery among institutional deliveries. Administrative region (division) was included as a random effect variable because there was a substantial difference in CS rate across different regions. We conducted likelihood ratio test for the randomness of the region variable and found the chi-square test statistic of 37.53 with significance level of <0.001. Therefore, inclusion of region as a random effect resulted in a better model predicting the factors of CS in Bangladesh. All analyses were performed using Stata version 13.1 [21].

## Results

### Characteristics of the mothers under study and who had caesarean section

Table 2 shows the descriptive statistics and the proportion of CS among different categories. Out of 4,627 mothers who delivered in health facilities, 1,122 (24%) delivered through CS. While most of the mothers belonged to 20–24 year (33.6%) and 25–29 year (25.7%) age brackets, there was no significant difference in CS rate across different age groups. Almost half of the mothers had secondary education (47.7%), and CS delivery is higher among the bachelor or higher educated mothers (56.9%) which are statistically significant. Similarly, more husbands had secondary education (31.8%); whereas, the CS was more prevalent among higher educated husbands (55.3%). Only 23.7% mothers reported to be employed in income-earning activities and CS was prevalent among mothers who were not employed (26.1%) which is statistically significant. Most of the husbands were farmers or workers (45.0%); while CS was conducted more for mothers whose husbands were professionals (57.6%; p<0.0001). Almost equal number of mothers watched TV for at least once a week and those who did not, but CS was conducted more for the mothers who had more exposure to media (36.0%; p<0.0001). Most of mothers reported their first pregnancy (39.9%) and they also experienced CS most (31.8%; p<0.0001). While most of the respondents lived in rural areas (73.9%), two-fifths (40.0%; p<0.0001) of the urban mothers underwent CS. Mothers belonged to richest wealth quintile also experienced CS more (53.5%; p<0.0001).

**Table 2:** Characteristics of the respondents and those who delivered by caesarean section (N = 4,627).

Characteristics	Number of respondents	% delivered by CS	*p-value (difference in proportions)*
	(%)N	(%)N	
**Total**	100.0 (4,627)	24.3(1,122)	
**Maternal age (years)**			
15–19	21.0 (971)	21.3(207)	0.4616
20–24	33.6 (1,554)	24.9(387)	
25–29	25.7 (1,189)	24.9(296)	
30–49	19.7 (914)	25.5(233)	
**Maternal Education (levels of schooling)**			
No education	14.1 (654)	7.5(49)	0.0001
Primary	28.0 (1,293)	12.5(161)	
Secondary	47.7 (2,208)	29.1(643)	
Bachelor or Higher	10.2 (472)	56.9(269)	
**Mothers currently working**			
No	76.3 (3,530)	26.1(920)	0.0002
Yes	23.7 (1,095)	18.4(202)	
**Husband’s education (levels of schooling)**			
No education	23.8 (1,103)	9(99)	0.0001
Primary	30.0 (1,386)	15.7(218)	
Secondary	31.8 (1,470)	29.6(436)	
Bachelor or Higher	14.4 (667)	55.3(369)	
**Husband occupation**			
Farmer/worker	45.0 (2,080)	13(270)	0.0001
Carpenter/mason/driver	24.2 (1,118)	26.9(301)	
Professional	5.9 (272)	57.6(157)	
Business	21.9 (1,012)	33.7(341)	
None/others	144(3.1)	36.9(53)	
**Watch TV at least once a week**			
No	50.8 (2,348)	12.8(302)	0.0001
Yes	49.2 (2,278)	36.0(821)	
**Birth order**			
First	39.9 (1,847)	31.8(588)	0.0001
Second	30.1 (1,393)	24.8(345)	
Third or higher	30.0 (1,387)	13.7(190)	
**Residence**			
Urban	26.1 (1,209)	40.0(483)	0.0001
Rural	73.9 (3,417)	18.7(639)	
**Wealth Index**			
Poorest	21.7 (1,002)	7.2(72)	0.0001
Poorer	18.9 (876)	11.1(97)	
Middle	19.1 (882)	19.7(174)	
Richer	20.6 (955)	30.5(291)	
Richest	19.7 (912)	53.5(488)	
**Mothers’s height**			
Less than 60 inches	40.1 (1,844)	25.5(469)	0.148
60 inches or more	59.9 (2,751)	23.3(641)	
**Mothers’s nutritional status**			
Undernourished	23.5 (1,090)	14.3(156)	0.0001
Normal	59.4 (2,747)	22.3(612)	
Overweight	16.1 (746)	45.7(341)	
**Size of the baby**			
Larger than average	12.9 (597)	30.6(183)	0.0267
Average	67.1 (3,106)	23.7(737)	
Smaller than average	20.0 (923)	21.9(203)	
**Number of antenatal care visits recieved**			
None	21.4 (991)	4.5(45)	0.0001
1–2	34.0 (1,575)	18.6(293)	
3+	44.4 (2,055)	38.1(783)	
**Antenatal care visit received from private sector**			
No	53.0 (2,451)	12.0(295)	0.0001
Yes	47.0 (2,176)	38.0(827)	
**Delivery in private facility**			
No	74.5 (3,445)	6.8(234)	0.0001
Yes	25.5 (1,180)	75.1(887)	

There was no significant difference in CS rate between mothers’ height categories of less than 60 inches and 60 inches or more. Most of the mothers were normal in terms of nutritional status (59.4%); whereas, CS was more prevalent among overweight mothers (45.7%; p<0.0001). Two-third of the babies was born with average weight, while 30.6% of the larger than average babies were born by CS, though it was not statistically significant. Most of the mothers (44.4%) received three or more antenatal care visits and almost half of them (47%) went to the private facilitates for the visit. More than one-third (38.1%) of those who received three or more antenatal care visits and 38% of those who visited private facility for antenatal care visits had CS (p<0.0001). While one-fourth (25.5%) of the mothers had delivery in private facilities, three-fourths (75.1%) of those who had delivery in private facilities had CS which is statistically significant.

### Mother-reported reasons for CS

Convenience and labor pain avoidance were two reasons which could contribute to the elective CS (CS not indicated by medical reasons); whereas, the other reasons pertain to the medical conditions compel to perform CS, e.g., malpresentation, premature baby, cord prolapse, labor obstruction, multiple births, pre-eclampsia, diabetes, previous CS, less pressure on baby’s body, other complications, and other reasons. Table 3 shows the reasons for choosing CS by principal decision makers, e.g., doctor or mother. In most of the cases (71.5%) doctors took the final decision for CS. Other complications were the principal reasons cited by mothers (29.9%) followed by malpresentation (20.9%), convenience (16.9%), and labor pain avoidance (15.1%). On the other hand, malpresentation was the major cause for the doctors (37.3%) followed by other complications (33.8%), failure to progress in labor (18.8%), and previous CS (13.9%) (Table 3).

**Table 3:** Reasons for choosing caesarean section for delivery by decision maker based on mothers’ report (N = 1,088).

Reasons	Mother side	Doctor side	Total
	% (n)	% (n)	% (n)
Convenience	16.9(53)	6.4(50)	9.4(103)
Avoid labor pain	15.1(47)	3.8(29)	7(76)
Mal presentation	20.9(65)	37.3(290)	32.6(355)
Premature baby	1.6(5)	2.3(18)	2.1(23)
Cord prolapsed	0.9(3)	2.3(18)	1.9(21)
Failure to progress in labor	13.5(42)	18.8(146)	17.3(188)
Multiple births	0.6(2)	0.3(2)	0.4(4)
Pre-eclampsia	1.8(6)	2.7(21)	2.4(27)
Diabetes	0.4(1)	0.7(5)	0.6(6)
Previous caesarean section	19.3(60)	13.9(108)	15.4(168)
Less pressure on baby’s body	2.8(9)	7.7(60)	6.3(69)
Other complications	29.9(93)	33.8(263)	32.7(356)
Others	4.4(14)	4.3(34)	4.4(48)
**Total**	**28.5 (311)**	**71.5 (777)**	**100.0 (1,088)**

### Timing of decision making on caesarean section

**Fig 1:**
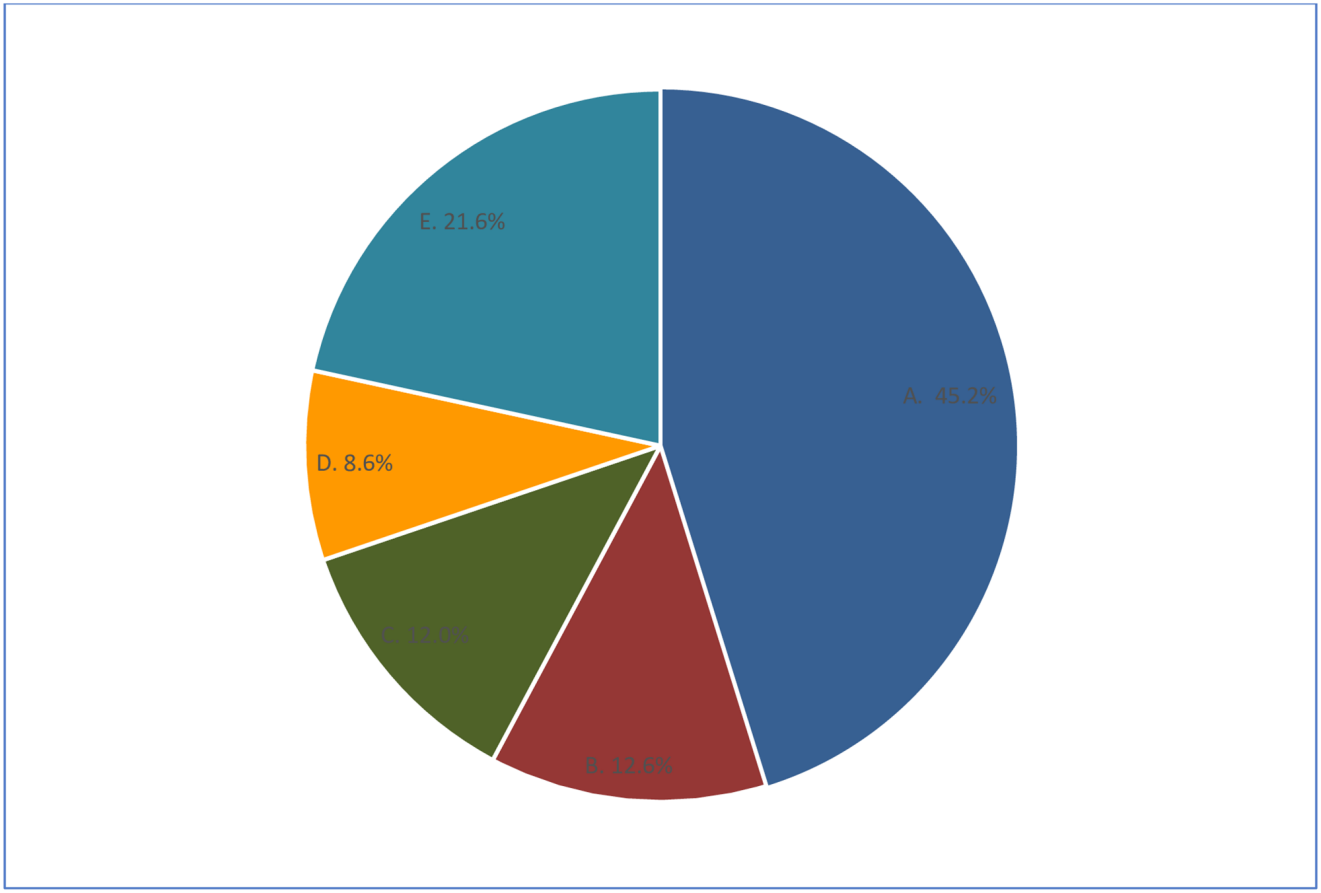
When decision was made for caesarean delivery, BDHS 2014. (A) Day of Delivery, (B) Day before Delivery, (C) 2 to 7 Days before Delivery, (D) 8 to 30 Days before Delivery, (E) 30+ Days before Delivery.

Timing of the decision for CS is showed in Fig 1. In most of the cases (45.2%) decision for CS was made on the day of delivery, while in one-fifth of the cases (21.6%) the decision was taken before one month of delivery date. Decision for CS was made on day before delivery in 12.7% cases, during the last week before delivery (2–7 days before delivery) in 12.0% cases, and between 8–30 days before delivery in 8.6% cases.

### Determinants of caesarean section delivery

Results from the multivariate analysis are furnished in Table 4. Older mothers aged 25–29 years and 30–49 years had higher odds of delivery by CS [OR = 2.29; CI = 1.55–3.38 and OR = 2.37; CI = 1.47–3.81 respectively] than adolescent mothers aged 15–19 years. Mothers who were employed had less odds of CS done [OR = 0.75; CI = 0.57–1.00] than who were not employed; while the mothers whose husbands are professionals had higher chance of getting CS done [OR = 1.62; CI = 1.00–2.64] than mothers whose husbands were farmers/workers. Chance of CS decreases with higher birth order, e.g., second birth order [OR = 0.58; CI = 0.43–0.78], and third order or higher [OR = 0.42; CI = 0.29–0.63] in comparison to the first birth. Mothers who lived in urban areas had higher odds of CS delivery [OR = 1.91; CI = 1.15–3.16] than mothers who lived in rural areas. Mothers who belonged to higher wealth quintiles had more chance of getting CS, e.g., middle [OR = 1.62; CI = 1.03–2.54], richest [OR = 1.98; CI = 1.18–3.32]. Mothers who were obese [OR = 1.63; CI = 1.14–2.32], and had larger than average sized baby [OR = 1.51; CI = 1.04–2.19] had higher odds of CS delivery. Mothers who received higher number of antenatal care visits from private facilities had higher chance of CS delivery, e.g., 1–2 visits [OR = 2.31; CI = 1.44–3.70], and 3 or more [OR = 3.47; CI = 2.18–5.52]. Mothers who had delivery in private facilities had higher chances of CS done [OR = 47.73; CI = 34.24–66.54]. If the delivery is conducted in an urban private facility the odds of it being a caesarean section are 50 times higher than it being a normal delivery.

**Table 4:** Association between caesarean delivery and selected socio-economic, demographic, biological, and institutional factors based on multilevel mixed effect model (N = 4,451).

Characteristics	Adjusted odds ratio	95% CI of OR	*p-value (OR is different from 1)*
**Maternal age (years)**			
15–19			
20–24	1.25	[0.91–1.7]	0.171
25–29	2.29	[1.55–3.38]	0.001
30–49	2.37	[1.47–3.81]	0.001
**Maternal Education (levels of schooling)**			
No education			
Primary	1.02	[0.63–1.63]	0.944
Secondary	1.26	[0.79–2.02]	0.336
Bachelor or Higher	1.47	[0.82–2.62]	0.196
**Mothers currently working**			
No			
Yes	0.75	[0.57–1]	0.050
**Husband’s education (levels of schooling)**			
No education			
Primary	0.98	[0.68–1.42]	0.925
Secondary	1.06	[0.72–1.55]	0.778
Bachelor or Higher	1.51	[0.94–2.45]	0.091
**Husband occupation**			
Farmer/worker			
Carpenter/mason/driver	0.99	[0.74–1.33]	0.948
Professional	1.62	[1–2.64]	0.050
Business	1.13	[0.84–1.51]	0.432
None/others	0.78	[0.41–1.47]	0.436
**Watch TV at least once a week**			
No			
Yes	1.07	[0.82–1.4]	0.635
**Birth order**			
First			
Second	0.58	[0.43–0.78]	0.001
Third or higher	0.42	[0.29–0.63]	0.001
**Residence**			
Urban	1.91	[1.15–3.16]	0.013
Rural			
**Wealth Index**			
Poorest			
Poorer	1.23	[0.78–1.94]	0.367
Middle	1.62	[1.03–2.54]	0.037
Richer	1.5	[0.94–2.39]	0.088
Richest	1.98	[1.18–3.32]	0.010
**Mothers’s height**			
Less than 60 inch			
60 or more	1.15	[0.93–1.44]	0.201
**Mother’s BMI**			
Undernourished			
Normal	1.13	[0.85–1.51]	0.390
Obese	1.63	[1.14–2.32]	0.007
**Size of the baby**			
Larger than average	1.51	[1.04–2.19]	0.028
Average	0.91	[0.69–1.21]	0.533
Smaller than average			
**Number of antenatal care visits recieved**			
None			
1–2	2.31	[1.44–3.7]	0.001
3+	3.47	[2.18–5.52]	0.001
**Antenatal care visit received from private sector**			
No			
Yes	0.85	[0.66–1.09]	0.193
**Delivery in private facility**			
No			
Yes	47.73	[34.24–66.54]	0.001
Delivery in private facility*Residence(Urban)	0.37	[0.24–0.56]	0.001

## Discussion

The study aimed to find out important socio-demographic and economic determinants of CS based on a latest nationally representative survey in Bangladesh. Facility delivery is promoted by the policy makers to decrease the maternal death because still three-fifths of all deliveries are performed at home and if complications arise the untrained or not-so-well trained delivery assistants are ill-equipped to perform life-saving maneuvers [22–25]. Despite a steady progress in increasing facility deliveries over the years, the meteoric rise in CS rate in facilities reflects the misuse of scarce healthcare resources and costs as well as poses great threats to mothers and newborns [5, 26]. In 2014, the all cause CS rate was 9% more in Bangladesh than WHO standard recommendation range of 10%-15% [2]. However, the rate is slightly higher than the lower minimum recommended level among mothers from the poorest quintile households and among mothers with no education. Different health financing mechanisms helped in contributing to the rapid rise of CS rates in some countries [27]. In Bangladesh the ongoing various maternal health program might be responsible for the increasing population based CS rates [16].

Mothers got the message that CS are convenient, less painful, and relatively easy which also creates the demand for elective caesarean section [28, 29]. In our study we can see that more than 16% mothers chose CS for convenience, and avoiding labor pain and when the patients are the principal decision maker these two reasons dominate. On the other hand, in most cases physicians went for CS when the pregnancies were complicated, e.g., malpresenttation of the fetus (37.3%), other complications (33.8%), and failure to progress in labor (18.8%). Physicians’ incentives for performing CS include both saving time from prolonged normal vaginal deliveries and making more money at the same time [30, 31]. The timing of the decision on CS also shows that decision on 42.1% CS was taken 2 or more days before delivery date. It signifies that a big proportion of CS was decided way before the indication for CS arises, e.g., complications.

Mothers who are educated, wealthy and reside in the urban areas have shown significant rise of CS rate which is quite alarming for a low- and middle-income country like Bangladesh where resources are scarce and problems are multiple. In Bangladesh some important social determinants of health, particularly female education have improved considerably during the last couple of decades, [32]. Results of this study indicate that educational gain was greater for mothers than their husbands. Studies in other settings have also explored the influence of maternal education on the use of maternity care services especially CS delivery [28]. In Bangladesh it can be said that with increasing urbanization, rise of average income and higher coverage of private facilities, the CS rate will continue to rise unless we take some strategic moves and impose some controlling provisions.

Higher percentage of mothers has taken more than one antenatal check-up indicating increase utilization of ANC services. Study results show that ANC services received from and deliveries performed in private facilities enhance the chance of CS. Private facilities which are mainly profit driven and have incentive to increase CS without proper indications for this procedure. In one of our neighboring countries (India) a similar situation exists where some states are expected to achieve high institutional delivery occurring in the private sector and majority of them will undergo CS procedure by 2020 [33]. Private health sector has seen a dramatic rise in Bangladesh during the last two decades [34] and maternal healthcare services occupies a significant part of it [35]. Several factors can contribute to the increase of CS delivery rate at private facilities. The important ones are perception of improved quality of care and accessibility of private facilities, availability of specialist physicians, a low trust level in the care provided by public facilities, the lack of availability of health services and drugs at public centers [36–38]. Previous reports’ findings suggested scarcity of qualified obstetrician/gynaecologists working in the public sectors is one of the main reasons for this rising utilization of private health facilities [39].

In case of low- and middle-income countries like Bangladesh actual motivating factors behind the increasing preference for caesarean section is still to be determined. It is argued that the physicians’ interests on the choice of CS holds a certain level of influence alongside the necessary medical factors [40]. In a study it was found that the physician’s expediency, fear of lawsuit, and most importantly, economic incentives may determine the choice of CS [41]. Another important element is the place of delivery. It was found that mothers who visited private facilities for antenatal checkup end up being delivered by CS [40]. Our findings confirm the results of earlier studies of the high prevalence of caesarean delivery in private facilities in other South Asian countries [42]. In India, a recent analysis of Annual Health Survey (AHS) data in nine States found that the median CS rate in the private sector was 28%, compared with 5% in the public sector [43].

Preference of the performance of CS depends on different medical emergencies that include high maternal age, obesity of mother, size of the baby, malpresentation of fetus, and failure to progress to labor [44]. These are the factors that are considered as risk factors and induce a preference for CS. Mother’s age also plays an important role in performance of CS. In this study mothers aged 30 or more have greater chances of CS delivery than younger mothers. Awareness developed due to increase exposure to different media like TV and due to increase socialization, mothers nowadays embark on pregnancy at a later age. Therefore, chances of CS are high for their delivery. Studies suggest that mothers are more prone to complications at later stage of their life [43]. We also found that rates of operative vaginal delivery were lower in the overweight group. The higher CS rates in the obese groups and the unwillingness of practitioners to perform vaginal deliveries in this population because of the increased risk of complications. In one study it was found an increased risk of shoulder displacement of the newborns in the overweight group [45]. Large size of baby at the time of delivery is another important factor for undergoing CS. Babies born later are less likely to be delivered by CS than first-borns and according to Mishra and Ramanathan [40] delivery complications are significantly lower among higher-order pregnancies.

In this study it was found that majority of CS was chosen by the mothers and their family as those had been suggested by doctors. Sometimes users are poorly informed of their health care needs and not surprisingly, unable to judge many aspects of quality and most importantly the costs of their health care need. Thus it ensues a logical query of whether delivery procedure of CS which is life-saving but significantly expensive surgeries are being conducted for the betterment of the mothers’ health and for their babies’ sake or it was mainly motivated by financial benefit of the service provider.

One of the limitations of the study is the recall period. In BDHS the recall period is three years and it is too long period to describe all events related to birth. In the survey, mothers were asked about the most recent live birth in the three years preceding the survey. However, the recall period was not always three years. In the sample, 36.8% of births happened in the previous year, 34.8% within 1–2 years and 28.4% beyond two years (2 to 3 years). Therefore, the possibility of recall bias should be quite low. Except for few questions almost all of the information collected in BDHS surveys is subject to reporting biases. Past events have been reported based on recall of the respondent in many cases such as date of birth of the children, the age of the respondent and antenatal care visit during pregnancy. This might inevitably causes biases. Most health information is provided by the women or based on women’s perception, like birth size of the baby. There may be a tendency of the mothers to report large size of the baby as a reasonable cause for undergoing CS delivery during the interview. Since the data do not contain actual measurement of the babies’ size, this might be also a limitation of our study. Since BDHS data is cross-sectional it does not allow doing the causal inference between covariates and outcome variable. However, the strength of this paper is that it used a very recent data to show the determinants of caesarean section.

## Conclusions

To keep the CS rate within expected level it is suggested that accreditation system of individual country should be strengthened and practiced [19]. Appropriate training, timely and regular supervision and leadership by senior physicians are also important in conducting standard practices. Another important thing that is highly suggested is to perform routine clinical audits. It would be very useful to perform routine clinical audits in facilities at all levels, which can be used to monitor the change of CS rate, improvement of practice and maintain a good quality of care [46, 47]. A proper guideline should be prepared in consultation with the obstetricians/gynaecologists and policy makers. Adherence to the guideline, along with enhance knowledge on the unsafe nature of the unnecessary CS can achieve increased institutional normal delivery in future; otherwise, an emergency procedure may end up being a lucrative practice.
